# Oculocutaneous albinism in southern Africa: Historical background, genetic, clinical and psychosocial issues

**DOI:** 10.4102/ajod.v11i0.877

**Published:** 2022-10-14

**Authors:** Jennifer G.R. Kromberg, Robyn Kerr

**Affiliations:** 1 Department of Human Genetics, Faculty of Health Sciences, University of the Witwatersrand and National Health Laboratory Service, Johannesburg, South Africa

**Keywords:** albinism and Africa, clinical management, culture, disability, epidemiology, genetics, genetic counselling, health, oculocutaneous albinism, psychosocial issues

## Abstract

Albinism is an inherited condition associated with significant depigmentation of the skin, hair and eyes. It occurs in every population with varying frequency, and narratives of people with albinism have been recorded since 200 BC. In southern Africa albinism is common, about 1 in 4000 people are affected, but it remains a poorly understood condition surrounded by myths and superstition. This article provides a historical background on oculocutaneous albinism (OCA) in southern Africa and presents relevant information from the literature regarding epidemiology, genetics and genetic counselling, health, psychosocial and cultural issues, and medical care. There are several recessively inherited types of OCA and a mutation, responsible for about 80% of South African variants, has been identified in OCA type 2. The physical characteristics associated with albinism, that is, sun-sensitive skin and low vision, can be managed. However, people with OCA in Africa also experience psychosocial issues, such as discrimination, because of the various superstitious beliefs and attitudes held in the community. Management should include medical care for health problems, appropriate adjustment of the schooling context and genetic counseling. In addition, widespread public awareness programmes are required to increase the knowledge of the genetic causes of OCA and of the nature of genetic counselling, to address the negative attitudes in the community, to reduce the marginalisation and stigmatization of people with albinism and to improve their quality of life.

## Introduction

Oetting et al. (1996) defined albinism as a

[*G*]roup of inherited abnormalities of melanin synthesis … characterised by a congenital reduction or absence of melanin pigment in association with specific developmental changes in the optic system resulting from the hypopigmentation. (p. 330)

The two main categories of the disorder are oculocutaneous albinism (OCA), which impacts the entire melanocyte system and causes hypopigmentation of the eyes, skin and hair, and ocular albinism (OA), which affects melanocytes of the eye with localised effects. The present article will focus on OCA, and the terms ‘person with albinism’ and ‘people with albinism’ will be used because those with the condition prefer these terms.

Albinism is a classic autosomal recessive Mendelian disorder caused by inherited gene mutations at a single gene locus, which results in a lack of pigmentation production by the cell (Rooryck et al. 2009). OCA occurs in every ethnic group at differing rates and is the type of albinism most often identified in African populations. The prevalence of OCA is higher in Africa than elsewhere, apart from rare, small and isolated populations found in Arizona (USA) and Panama (Witkop et al. 1972).

The aetiology of albinism is often misunderstood in Africa and is associated with many myths and superstitions. These common beliefs interfere with the normal development and social interactions of those affected and lead to stigmatisation and discrimination in the community (Kromberg 2018a); these beliefs also result in violations of the human rights of people with albinism (Reimer-Kirkham et al. 2020). For these and other reasons (mainly the associated physical disorders, such as low vision), the international community and the World Health Organisation (WHO) have declared albinism a disability (Clarke & Beale 2018).

At present, the condition is receiving attention in many health departments and disability services across Africa (Ero 2020). Therefore, the objective of this paper is to discuss oculocutaneous albinism in the black African population, with specific reference to published reports on the historical background, albinism as a disability, epidemiology, genetics and genetic counselling, health, psychosocial and cultural issues, and management of the condition. Following the human rights violations against individuals with albinism in Africa (discussed at https://www.underthesamesun.com/, accessed 22 February 2022), such a report is required to provide health care professionals with a comprehensive view of OCA and enable them to advocate on behalf of affected individuals in their institutions and communities.

## Historical background

Reports on people with albinism have appeared in the literature for many centuries, and to put the condition in context, selected publications will be briefly reviewed. The earliest description appears in the ancient Pseudoepigrapha (written around 200 BC and discussed in Sorsby 1958). This report states that Noah, at birth, had skin white as snow, white hair and eyes that shone like the rays of the sun. As Sorsby (Professor of Ophthalmology at Oxford University) explained, these features were very unusual in the Arab community into which Noah was born. Also, Noah was the son of first cousins and could have inherited a recessive gene mutation from both parents, resulting in his albinism.

Another very early report, from the first century AD, was written by Pliny the Elder, a Roman author and natural philosopher (Plinius Secundus the Elder 1942 translation). He stated that he had read a document describing men in Albania who had sea green eyes, white hair from childhood and difficulty seeing in the daylight. Pliny also described a group of Leucoethiopes (white Ethiopians) living in North Africa. Much later, Vossius, writing in 1660, stated that these people were also called albini (Pearson, Nettleship & Usher 1913).

In the 1850s, David Livingstone (1857), the Scottish missionary and explorer, described in detail a case of albinism he encountered in Botswana. He stated that the mother refused to kill her son who had albinism (as her community expected her to do), so she was excommunicated from her village. She and her son survived in isolation for many years but eventually she decided she had to kill him so that she could return to her village life.

In the early 20th century, Pearson and his colleagues collected information on albinism from many different countries and published their findings, with many photographs, in several volumes of their ‘A Monograph on Albinism in Man’ (Pearson et al. 1913). This most informative source described the physical signs and prevalence of the condition, as well as the psychosocial and cultural issues that surrounded it and the ways in which affected people were treated.

Further studies were conducted in Africa. Barnicot (1952) studied albinism in Nigeria and found that 1 in 2858 school children had albinism; some had unusual signs of the condition, with darker hair and more tanned skin colour, whilst others had red hair. Cohen et al. (1952) found that skin cancer in people with albinism in South Africa was common, particularly on the exposed areas of the body. Sun-barrier creams, hats and suitable clothing were necessary to prevent the development of cancer. Oettle (1963) documented cases in the Transkei (Eastern Cape Province, South Africa) and found the prevalence rate was 1 in 3759. Later, Wright, Norval and Hertle (2015) reviewed skin sensitivity, photoprotection and albinism, highlighted the challenges for at-risk individuals and made suggestions for how these could be resolved.

In the early 1970s, the staff of the Department of Human Genetics at the University of the Witwatersrand began a series of studies on albinism (initially supported by South African Medical Research Council grants) that continued for five decades. Studies covered epidemiological, clinical, psychosocial and scientific aspects of the condition, and information increased significantly. Many theses and papers were written and a comprehensive book, ‘Albinism in Africa: Historical, Geographic, Medical, Genetic and Psychosocial Aspects’ (eds. Kromberg & Manga 2018) was published.

## Albinism and disability

The marginalising (treatment of a person as insignificant or peripheral; Oxford English Dictionary) of people with albinism has been an ongoing issue over the centuries, particularly in Africa (Pearson et al. 1913), but publications have focused on this topic only recently. Clarke and Beale (2018) analysed the meaning, background and causes of marginalisation, the challenges it creates, and possible solutions to these issues. Marginalisation is inextricably intertwined with stigmatisation, discrimination and human rights abuses, which in the case of albinism has led to the condition being classified as a ‘socially produced disability’ (Clarke & Beale 2018:257).

Prior to the declaration of albinism as a disability in 2013, the suggestion that albinism was a disability was contentious. The visual impairment associated with the condition can often be treated (though seldom completely corrected), and skin sensitivity is manageable with the proper use of sun-barrier creams, sun avoidance (where feasible), long-sleeved cotton clothing and hats. Therefore, using the term disability in connection with albinism could be considered inappropriate. However, it is accepted that disability is not only medically determined but can also be socially produced (Clarke & Beale 2018). In 2006, the interaction between a person’s physical impairments and the society in which he or she lives was emphasized in the Convention on the Rights of Persons with Disabilities. In Africa, persons with albinism are more visibly different from their peers than they are in Western societies, where populations are mostly pale in skin colour. In addition, albinism in Africa is surrounded by misunderstandings, prejudice, discrimination, and ostracism (Imafidon 2019). As a result, the ‘disability rights approach’ is appropriate when facing challenges and finding solutions concerning the rights and equality of persons with albinism (Possi & Possi 2017). As these authors (2017) conclude:

[*T*]he task ahead is to ensure that the needs of persons with albinism are exhaustively addressed in future policies and programmes so as to cater for the full realization of their rights. (p. 140)

Since the WHO has recognized albinism as a disability, the United Nations Human Rights Council has appointed an Independent Expert (Ms I Ero) to monitor the Human Rights issues of people with albinism (Ero 2020). She has visited many African countries to bring these issues to the attention of governments and health departments. In February 2020, she reported to the 43rd session of the Human Rights Council, on her visit to South Africa. She commented on challenges regarding health, discrimination, education, employment, access to justice and harmful practices. She also described the community awareness initiatives that were developing and the national action plan, linked to the Regional Action Plan on Albinism in Africa and adopted by the African Union.

## Epidemiology

Estimates of the prevalence rates of albinism range widely throughout the world. However, the few available studies on epidemiology vary in quality and reliability and in some cases are based on small, inadequate samples. A worldwide rate of 1 in 17 000 was suggested by Witkop, Quevedo and Fitzpatrick (1983), and although this is probably outdated, no new estimates have been published. In Europe, estimates range from 1 in 10 000 in Ireland (Froggatt 1960) to 1 in 15 000 in the Netherlands (van Dorp 1987). Although, one state surveyed in the USA had a total prevalence rate of 1 in 17 000, the rate for the black population was 1 in 10 000, whilst that for the white population was 1 in 19 000 (Witkop et al. 1983). The rates in two small geographical isolates were 1 in 227 and 1 in 213, in the Hopi people of Arizona and the Cuna people of Panama, respectively. The condition appears to be rarer in Asia, and an estimate for the Chinese Han population was 1 in 18 000 (Gong et al. 1994), whilst in a Japanese population it was 1 in 47 000 (Neel et al. 1949).

The available statistics from the few published sub-Saharan African studies, with large samples and comprehensive ascertainment methods, are all greater than 1 in 5000 (Kromberg 2018b). Some examples of the rates of albinism in South Africa and its neighbouring countries are summarized in Table 1.

**TABLE 1:** Prevalence rates of albinism in Southern African countries.

Country	Rate	Size of sample	Reference
Botswana	1 in 1307	18 300 (Tswana isolate)	Kromberg 1985
Namibia	1 in 1735	2 113 077 (Census population)	Lund & Roberts 2018
South Africa	1 in 3900	803 511 (Soweto)	Kromberg & Jenkins 1982
Swaziland	1 in 1951	160 000 (Hhohho district)	Kromberg 1985
Zimbabwe	1 in 4720	1 312 032 (school children)	Lund & Roberts 2018
Zimbabwe	1 in 1000	11 000 (Tonga isolate)	Lund et al. 1997

*Source:* Adapted from Kromberg, J.G.R., Manga, P. & Kerr, R., 2020, ‘Children with oculocutaneous albinism in Africa: Characteristics, challenges and medical care’, *South African Journal of Child Health* 14(1), 50–54. https://doi.org/10.7196/SAJCH.2020.v14.i1.1608

The higher rates occurring in Africa could be maintained by selective advantage of the carriers of a gene for albinism, if they are lighter in skin colour (as suggested by Oettle 1963 and confirmed by Kromberg 1985) and preferred as marriage partners, and/or by mating patterns (such as preferences for consanguineous mating).

## Genetics of oculocutaneous albinism and genetic counselling

Oculocutaneous albinism is inherited as an autosomal recessive condition, and therefore it is advisable for every family with a member with albinism to have genetic counselling. An individual can only have albinism if he or she has inherited two mutated OCA genes, one from the father and one from the mother. Genetic counsellors can explain to the family that if an individual is affected, both parents must be carriers. and even though they themselves have normally pigmented skin, they are referred to as ‘obligatory’ carriers of a gene mutation. Because affected individuals have two non-functional OCA genes, pigment cannot be made and skin, hair and eyes appear pale in colour from birth onwards. Both males and females are equally affected. Once a couple has had a child with OCA, they will know that they are both carriers and will have a 1 in 4 (25%) chance of having another child with OCA; this is the same chance for every pregnancy (Harper 2004). Also, because the parents are carriers, their other children who do not have OCA can be carriers (with a 66% risk) and the parents’ brothers and sisters can be carriers too (with a 50% risk). The inheritance pattern of OCA follows the basic rules of Mendelian autosomal recessive inheritance. For a more comprehensive explanation of inheritance patterns and risk calculation, see (http:/www.genetics.edu.au/publications-and-resources/facts-sheets).

People with OCA pass on only one of their two albinism genes to their children, so all these children will be ‘obligatory’ carriers (Kromberg 2018c). If the person with OCA marries a carrier, then half (50% or 1 in 2) the children, who by chance inherit the gene from both parents, will have albinism. If couples have questions on the genetics of OCA, they can attend a genetic counselling clinic.

Presently, seven types of OCA (each determined by mutations in a different gene) have been described. Only three of these have been reported in Africa (Kromberg et al. 2012), namely OCA types 1, 2 and 3:

- In *OCA1*, the person with albinism has very pale skin, hair and eyes (usually blue); skin cancer risk is high; and vision usually poor throughout life. This type is very rare in Africa, and so far only one case has been described (in Cameroon) and confirmed by molecular testing (Badens, Courrier & Aquaron 2006).
- In *OCA2*, the person has pale skin, hair and light brown or blue eyes; the skin and hair can darken a little with age and begins to become yellowish in appearance. Dark patches or freckles might develop on the skin, cancer risk is lower and vision a little better than in *OCA1*. *OCA2* is by far the most common type of albinism seen in Africa.
- In *OCA3*, or rufous albinism, the person has pale reddish skin, fair to ginger hair, dark blue to brown eyes, hair and skin can darken with age, and skin cancer risk is lower and vision is better than in *OCA* 1 and 2; gene mutations have been found in the *TYRP 1* gene on chromosome 9 (Manga et al. 1997). It is estimated that in the black population, about 1 in 8500 individuals have *OCA3* in South Africa (Kromberg et al. 1990).

Nearly all cases of albinism in African individuals are caused by mutations in the *OCA2* gene (previously referred to as the *P* gene) which maps to the long arm of chromosome 15. Further, a common *OCA2* mutation has been described in southern Africa; this mutation accounts for 78% of all disease-causing mutations in this population, and about 1 in 30 black southern Africans carry this mutation (Stevens et al. 1995). The mutation is a deletion (2.7 kb in size) within the *OCA2* gene (Durham-Pierre et al. 1994). Diagnostic genetic testing for this mutation is available through the National Health Laboratory Service in South Africa.

## Clinical considerations

If an infant has albinism at birth, it is often obvious, especially in dark-skinned populations. The pale skin and hair colour are usually significantly different from that of the parents (Kromberg, Zwane & Jenkins 1987). Within a few days, eye problems become apparent, and nystagmus (fast, generally horizontal movements of the eyes), lack of focus and photophobia (increased sensitivity to light) are evident (Kammer 2018). Sun-sensitive skin and low vision, however, are the major health issues.

The skin of people with albinism is highly sensitive to the sun because it has little or no melanin pigment to act as a barrier to the harmful effects of the ultraviolet rays (Hartshorne & Manga 2018). In one local study, 23% (25/111) of persons with albinism (aged 1–60 years) had solar damage, skin lesions and/or keratoses, and damage increased with age (Kromberg et al. 1989). In the group of 1- to 19-year-old participants (*n* = 76), 20% (15/76) already had sun damage. The face, cheek and eye lids were the most commonly affected sites, and squamous cell carcinoma was the most frequent type of cancer diagnosed. To prevent damage, the skin must be protected from a very early age. As soon as the child can go out in the sun, anti-actinic sun-barrier creams (SPF 50) must be applied daily (and lifelong), particularly on the exposed areas of the skin. Also, sun exposure from 10:00 to 15:00 should be avoided, and cotton clothing with long sleeves and trouser legs as well as hats with large brims should be worn regularly (Hartshorne & Manga 2018).

Visual problems, ranging from mild to severe, occur in all those with OCA. The majority of affected people have nystagmus and photophobia and about 30% have strabismus (squinting) (Kammer 2018). Visual defects can result in reduced visual acuity, refractive errors and myopia, poor stereoscopic, binocular and in-depth vision. To manage these health issues, regular clinical assessment at an eye clinic is essential, starting in the first year of life. Many refractive errors can be corrected with spectacles, but vision cannot be restored to normal levels. The use of dark glasses helps with photophobia.

Intelligence in people with albinism is within the normal range (Manganyi, Kromberg & Jenkins 1974). Therefore, education in regular community schools is recommended. However, teachers need to be informed about the condition so that they allow the affected child to sit at the front of the class (because of the low vision), away from glare, and wear a hat if necessary (Kammer 2018). Also, teachers need to provide enlarged printed wording in school notes where possible and permit a longer time to be taken on assignments and examinations. Portable hand magnifiers and telescopes are also useful. Children with OCA can then cope with a little extra help from the teacher.

## Psychosocial and cultural issues

Various psychosocial issues arise as soon as a child with albinism is born (Kromberg et al. 1987). Nurses may not understand the cause of the condition, may not want to touch the infant for fear of infection and may give the mother little or no accurate information. The mother herself may be shocked and upset; initially she may become depressed and reluctant to hold, feed and care for the infant, and (rarely) may abandon the child. However, after three months, the mother has usually accepted her child, adapted and maternal–infant bonding has developed. The father may have similar reactions to those of the mother unless he has a family history of OCA; however, some fathers deny paternity and a few abandon mother and child. Other members of the family may also be shocked and unwilling to accept the child, and if they do, they might be stigmatized in the community (Kromberg 2018a). Munyare (2004), writing on his personal experiences in Kenya, states:

My birth was very traumatizing to my entire family because it was hard to convince the entire community that I was one of them. (p. 31)

Adjustment is often difficult for the growing child as well as for teenagers and adults with albinism. Hernandez and Harper (2007) reviewed the personal and psychological aspects of albinism and concluded that persons with albinism face physical, social, psychological and emotional challenges. The social impact of the visible difference associated with their condition is difficult to manage, and personality problems, including low self-esteem, poor coping and social skills, may result. Further, the development of social identity and self-concept may be problematic, whilst community groups tend to ostracise and reject individuals with albinism. Biesecker and Erby (2008:402) suggest that adaptation to living with a genetic condition requires ‘struggling effectively, working through learning for adversity and integrating the experience into one’s life’, and they added that most human beings are resilient and learn to cope; however this view might be over-simplistic.

Stigmatisation is common where people with a disability or people who differ in some way from their peers come into contact with community members. If a condition is rare and the community has not had any experience of it, nor of interacting with affected people, discrimination can occur. Wan (2003) states that most of the prejudice towards people with albinism is because of a fear of the unknown; such fearful feelings giving stigma its power and reality. However, the myths and superstitions that surround the condition in African communities are much more of a stumbling block than the actual physical condition.

Attitudes towards people with albinism range from negative to extremely positive. Negative attitudes have led to rejection and even infanticide, whilst positive attitudes have resulted in the individuals being considered lucky, favoured as doctors or sorcerers, or sacred and under divine protection (Pearson et al. 1913). Even where communities are familiar with the condition and generally accept people who have albinism, acceptance can stop short of marriage (Kromberg & Jenkins 1984). Attitudes were, and still are, affected by the widely held myth that people with albinism do not die, but disappear. The belief in this myth unsettles the community, as well as people with albinism (Kromberg & Jenkins 1992). The origin and development of the myth, and why it is maintained in the community, is poorly understood. Baker and Djatou (2007) suggest that it may be associated with the marginalised nature of albinism as neither truly white nor black or the intermediate state of the condition and its position between two worlds (the real and the spiritual, or possibly even the living and the dead).

Recently, a myth, with more serious implications, has gained strength. This myth promotes the belief that medicine made from the body parts of people with albinism is powerful and effective in bringing good luck to the user (Mostert & Weich 2017). Consequently, people with albinism have been abducted, murdered and mutilated in several countries in Africa over the last decade. Recent figures show that between 2006 and 2017, about 190 murders and 515 attacks were reported in 27 African countries (Clarke & Beale 2018). This myth has origins in the past when body parts, particularly the skin, of deceased chiefs were used to make medicines for the new chief to bring him power and success in rainmaking (Eiselen & Schapera 1937).

The outcome of both the physical and the psychosocial factors associated with having albinism is that the quality of life of those affected is compromised. In a study on psychosocial issues conducted in Malawi, the results showed that the stigmatization and discrimination people with albinism face made life difficult for them (Braathen & Ingstad 2006). New social situations were particularly hard to manage, but once others found the person with albinism was not so different from themselves, acceptance improved. Similarly, further recent research findings from Malawi show that people with OCA suffer socially because of negative community beliefs and public misunderstandings (Tambala-Kaliati et al. 2021). Another research group investigated quality of life in Brazil and reported that the social reality of people with albinism needed more attention because the social segregation and myths they experienced had both medical and psychosocial implications (Maia et al. 2015). These researchers added that the prejudice faced by affected people could result in emotional instability and less assertiveness and, therefore, a reduced quality of life. In addition, vision-specific factors affect many aspects of daily living and functioning, as well as quality of life; people with albinism have reported problems with vision-specific roles, distance acuity and mental health (Kutzbach et al. 2009).

## Recommendations for management

Soon after the birth of a child with albinism, parents should be referred for genetic counseling so that they understand the cause of the condition, how to cope with it and how to explain it to others (Kromberg 2018c). This encounter should cover inheritance and recurrence risks; prognosis; physical, psychosocial and cultural aspects; and how best to manage health and education challenges (Kromberg & Jenkins 1984). As Taylor et al. (2021:1) state, ‘a biomedical explanation helps to establish a baby with albinism as a real person with a genetic difference’, fostering greater acceptance.

The child should then be referred for dermatology and ophthalmology assessments in the first year of life. The parents need to be made aware that to assist with low vision, the child should have regular assessments and appropriate visual aids (Kammer 2018). Also, to prevent sun damage, the child should have an annual skin examination, apply sun-barrier cream (SPF 50) daily to exposed parts of the body, wear appropriate cotton clothing and avoid sun exposure whenever possible (Hartshorne & Manga 2018). Later, both parents and children will benefit by having psychological counselling to encourage adaptation to the condition. If the children are nurtured, regarded as worthwhile individuals and raised to be assertive, independent and self-confident, they can strive to reach their potential rather than succumb to society’s negative attitudes (Ezeilo 1989).

To minimize stigmatisation and improve the chances that people with albinism become integrated into their communities, ongoing public awareness programmes should be initiated (Tambala-Kaliati, Adomako & Frimpong-Manso 2021). Such campaigns have been offered by Standing Voice, an international non-governmental organization (NGO) operating in Tanzania and defending the rights of persons with albinism, and other NGOs working in Africa (Clarke & Beale 2018). Ideally these programmes should be introduced at many levels of society, and targeted at health professionals, who will generally be the first people the mother encounters after the birth, as well as at teachers, the general public and the families themselves, who often need to advocate for their affected member. The message needs to include the fact that albinism is inherited and caused by mutated genes; many unaffected people are carriers; intelligence in affected people is in the normal range; their death is the same as that of any other person; their body parts can never make powerful medicine; the common myths are untrue; and, lastly, that albinism is a manageable condition, when properly treated.

Further research is also required and the quality of life in both adults and children (Taylor, Bradbury-Jones & Lund 2019) with OCA should be investigated. Ero (2020) has recommended that more data on health discrimination, education, employment and access to justice and harmful practices should be collected. In addition, a detailed situation (both rural and urban) analysis of people living with OCA is necessary.

## Conclusion

Many people are familiar with the sight of a person with OCA, especially in Africa. However, as this report shows, their unusual appearance, as well as the superstitions that surround the condition, can result in marginalisation, stigmatisation, rejection and a poor quality of life. Good health and development are possible for people with albinism who have their needs met, receive adequate health care from trained health professionals, educational opportunities from informed educationalists and support from their families. In addition, if the community in which they live receives appropriate information, and if awareness increases, the myths and superstitions can be debunked, prejudice can be reduced and the quality of life for people with OCA can be improved.
